# Fracture Behavior of Basalt Fiber-Reinforced Airport Pavement Concrete at Different Strain Rates

**DOI:** 10.3390/ma15207379

**Published:** 2022-10-21

**Authors:** Yifan Mu, Haiting Xia, Yong Yan, Zhenhui Wang, Rongxin Guo

**Affiliations:** 1 Yunnan Key Laboratory of Disaster Reduction in Civil Engineering, Faculty of Civil Engineering and Mechanics, Kunming University of Science and Technology, Kunming 650500, China; 2 Faculty of Civil Aviation and Aeronautics, Kunming University of Science and Technology, Kunming 650500, China

**Keywords:** airport pavement concrete, basalt fiber, fracture parameters, strain rate

## Abstract

As a commonly used surface structure for airport runways, concrete slabs are subjected to various complex and random loads for a long time, and it is necessary to investigate their fracture performance at different strain rates. In this study, three-point bending fracture tests were conducted using ordinary performance concrete (OPC) and basalt fiber-reinforced airport pavement concrete (BFAPC) with fiber volume contents of 0.2, 0.4, and 0.6%, at five strain rates (10^−6^ s^−1^, 10^−5^ s^−1^, 10^−4^ s^−1^, 10^−3^ s^−1^, and 10^−2^ s^−1^). Considering parameters such as the peak load, initial cracking load, double K fracture toughness, fracture energy, and critical crack expansion rate, the effects of the fiber volume content and strain rate on the fracture performance of concrete were systematically studied. The results indicate that these fracture parameters of OPC and BFAPC have an obvious strain rate dependence; in particular, the strain rate has a positive linear relationship with peak load and fracture energy, and a positive exponential relationship with the critical crack growth rate. Compared with OPC, the addition of basalt fiber (BF) can improve the fracture performance of airport pavement concrete, to a certain extent, where 0.4% and 0.6% fiber content were the most effective in enhancing the fracture properties of concrete under strain rates of 10^−6^–10^−5^ s^−1^ and 10^−4^–10^−2^ s^−1^, respectively. From the point of view of the critical crack growth rate, it is shown that the addition of BF can inhibit the crack growth of concrete. In this study, the fracture properties of BFAPC were evaluated at different strain rates, providing an important basis for the application of BFAPC in airport pavement.

## 1. Introduction

At present, cement concrete is the most common material for airport runway surfaces. With increased traffic volumes, airport pavement disease has become increasingly evident, with the appearance of cracking, settlement, cavities, and other problems during its operation. In order to solve the pavement concrete cracking problem, the application of fiber-reinforced concrete for airport road surfaces has been extensively studied. Chen et al. [1,2] studied the effect of various fibers on the strength and durability of airport pavement concrete. It was found that the flexural strength was increased by 6% when modified polyester fibers (MP) and polyacrylonitrile synthetic fibers (PS) were blended into concrete. Under a high temperature aging environment, 1.5% steel fiber (SF) significantly inhibited the aging cracking and effectively improved the wheel impact resistance of airport pavement concrete. The addition of PS and MP led to the greatest enhancement effect on impermeability and frost resistance, respectively. Merhej et al. [3] analyzed the effects of polypropylene fibers (PP) on the compressive strength, modulus of rupture, load–deflection curve, and flexural toughness of airport pavement concrete. In light of the fatigue failure of airport pavement concrete, Wang et al. [4] investigated the impact of polyoxymethylene fiber (POM) on the flexural fatigue performance of airport pavement concrete. Based on the above studies, it has been well-demonstrated that fiber-reinforced concrete has broad application prospect for the construction of airport roadway surfaces.

Basalt fiber (BF) is a kind of material with high tensile strength and high elastic modulus. Different from other fibers, BF has the characteristics of high-temperature resistance, acid and alkaline resistance, ease of dispersion, and low price [5,6]. Concrete mixed with a certain volume fraction of BF can effectively improve its cracking, bending, and impact strength, while reducing the pore size of concrete and refining the pore structure [7,8,9,10]. In addition, scholars have found that basalt fiber-reinforced concrete (BFRC) has excellent fracture properties. Wang et al. [11] investigated the fracture properties of BF-reinforced fly ash geopolymer concrete (FAGC) and found that the incorporation of BF can significantly improve the fracture energy and fracture toughness of FAGC. BF can play an important role in inhibiting the crack propagation of concrete. Smarzewski [12] found, in a three-point bending fracture test of high-performance basalt fiber-reinforced concrete (HPBFRC), that with an increase in fiber content, the fracture energy of the specimens increased gradually, presenting a positive linear relationship. Arslan [13] has stated that the addition of BF and glass fiber (GF) into concrete both improved its flexural strength, ductility, and fracture energy. It was found, through scanning electron microscopy (SEM), that the addition of BF enhanced the bonding properties between the substrates. Based on the above summary of BFRC performance, it can be inferred that BF also has a good application prospect in airport pavement [14]. Sun et al. [15] have considered the problem of airport pavement cracking and durability deficiency, and the application of basalt fiber airport pavement concrete (BFAPC) was studied through a static mechanical test and numerical simulation. At present, the airport’s operational environments are complex. Airport pavement is not only affected by the static load of aircraft, but also by dynamic impact loads caused by pavement roughness and aircraft landings [16,17]. As airport pavement concrete is prone to fracture under dynamic loads, and the subsequent damage and deterioration can greatly affect the safe takeoff and landing of the aircraft, it is necessary to study the fracture behavior of airport pavement concrete under dynamic loads.

Concrete is a rate-sensitive material with more complex fracture patterns at high loading rates. Many scholars have investigated the fracture properties of materials at different loading rates. Xuan et al. [18] analyzed the effects of different loading rates on the fracture properties of steel fiber cement matrix composites. It was shown that the initial cracking load, unstable fracture toughness, and fracture energy increased in different magnitudes with an increase in the loading rate. Chen et al. [19] conducted fracture tests on concrete at different loading rates based on acoustic emission (AE) technology and found that the peak load and fracture toughness increase with the increase in loading rate. Chen et al. [20] analyzed the effect of different loading rates on the fracture properties of concrete using three methods: A three-point bending test, digital image correlation (DIC), and ABAQUS finite element method. They found that the fracture toughness and fracture energy of concrete at low loading rates have a significant rate effect. Ma et al. [21] concluded that the fracture toughness of concrete is linearly related to the logarithm of the strain rate, and the length of fracture process zone (FPZ) increases with an increase in loading rate. The above studies have shown that the loading rate has a significant effect on the fracture parameters of concrete, but the fracture performance of BFAPC at different strain rates has been less studied. In addition, it is necessary to monitor the crack extension changes during the fracture process. DIC is a non-destructive testing method, which can be effectively applied to study concrete fracture performance [11,22,23]. It can be used to analyze the changes of displacement field and strain field in the process of concrete fracture with high accuracy. Crack extension changes can be quantified by DIC, and crack extension during the fracture process is affected by the crack tip displacement field [24,25]. For fiber-reinforced concrete, if the addition of fiber can reduce the crack extension length of concrete under the same load level, it indicates that fiber can inhibit crack growth [11,22].

In summary, most scholars have studied fiber-reinforced airport pavement concrete in terms of strength and durability. Various types of loads, such as taxiing, takeoff, and landing, are frequently exerted on airport pavements, and concrete is a rate-sensitive material that exhibits different fracture properties at different strain rates. Therefore, from the perspective of how the strain rate affects the fracture performance of BFAPC, its application value in airport pavements can be further reflected.

In this paper, based on fracture mechanics theory, three kinds of BF content (0.2%, 0.4%, and 0.6% volume fraction) and five kinds of strain rates (10^−6^ s^−1^, 10^−5^ s^−1^, 10^−4^ s^−1^, 10^−3^ s^−1^, and 10^−2^ s^−1^) were considered, according to the strain rate value range under different working conditions. The three-point bending fracture test of a pre-cast crack airfield pavement concrete beam was carried out, and DIC was used to monitor the crack growth process during the test. The rate effects of BFAPC fracture parameters at different strain rates were analyzed, in terms of peak load, initial cracking load, double-K fracture toughness, fracture energy, and critical fracture extension rate.

## 2. Materials and Methods

### 2.1. Raw Materials and Mix Proportion

**Figure 1:**
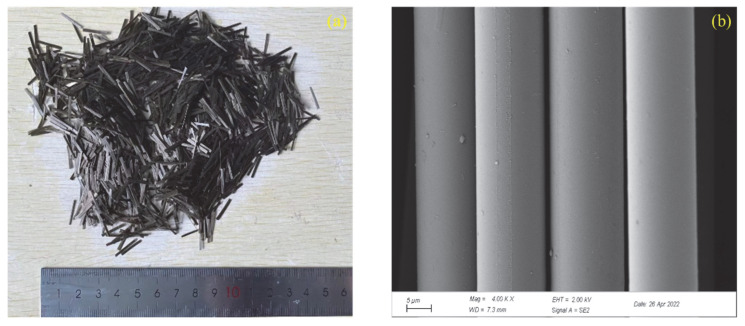
(**a**) Macro and (**b**) micro morphologies of BF.

According to the Specifications for Airport Cement Concrete Pavement Design (MH/T 5004–2010) and concrete strength design requirements, the mixing ratio of ordinary performance concrete (OPC) is shown in Table 1. In the label “B-J”, “B” represents the BFAPC specimen, and “J” represents the fiber volume content. The selected materials were ordinary Portland cement (P·O 42.5, density 3.10 g/cm^3^), fly ash (F-type I low-calcium, density 2.30 g/cm^3^), granulated blast furnace slag powder (S95-grade, density 2.90 g/cm^3^), polycarboxylic acid high-performance water reducing agent (CNMB Zhongyan Technology Co., Ltd., Changsha, China), coarse aggregate (two continuous gradings: 4.75–16 and 16–30.5 mm), fine aggregate (machine-made sand, fineness modulus 3.2), and basalt fiber (Shanghai Chenqi Chemical Technology Co., Ltd., Shanghai, China). Figure 1 shows the macroscopic and microscopic morphology of BF, with a length of 12 mm. Table 2 displays its physical–mechanical indices.

### 2.2. Specimen Preparation

Concrete with dimensions of 400 × 100 × 100 mm was prepared according to the mixing ratios in Table 1. A steel piece was pre-buried at the mid-span position before casting. The surface of the steel piece was coated with lubricant, in order to facilitate the formation of pre-fabricated cracks. In the study by Zhao et al. [26], it was shown that the crack–height ratio often has a certain impact on the K-dominant zone (the applicable region of linear elastic fracture criterion) at the front end of the cracks. When the crack–height ratio was 0.4, the T-stress (a constant term in the series expansion of crack tip displacement field)—which has a significant effect on the stress field around the crack tip—was close to 0, and the calculated fracture toughness was more reliable. Therefore, the crack–height ratio (*a*_0_/*h*) was set as 0.4. The pre-fabricated fracture depth *a*_0_ was 40 mm, the fracture width was 3 mm, and the fracture tip was 15°. The BF was added into fresh concrete at 0.2%, 0.4%, and 0.6% volume content to prepare BFAPC samples. For a better dispersion effect, the fiber was added through a dry mixing method: sand and stone were first mixed with part of the fibers in the mixer for 60 s, following which the cementitious material and the remaining part of the fibers were added for 60 s. The water and water reducer mixture was slowly added and mixed for 2 min. Finally, the concrete mix was poured into the mold, properly vibrated to ensure that the mix was dense, and the specimens were covered with cling film. The steel piece was removed after 6 h, demolded after 24 h, then sent to a standard curing room with a temperature of 20 ± 2 °C and relative humidity greater than 95% for curing (with an age of 28 d).

### 2.3. Test Method

**Figure 2:**
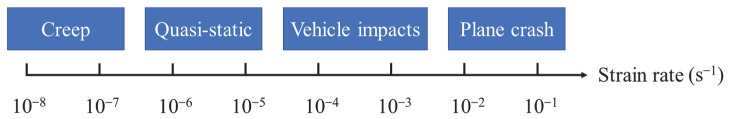
Regimes of strain rates.

In the test, the specimen was subjected to three-point bending loading using an MTS-810 testing machine (MTS Systems Corporation, Eden Prairie, MN, USA). The span S was 300 mm, and the loading mode was controlled by displacement. In order to compare the fracture damage of concrete beams at different strain rates, Figure 2 displays the range of strain rates under different working conditions [27,28]. The loading rates were 0.009 mm/min, 0.09 mm/min, 0.9 mm/min, 9 mm/min, and 90 mm/min, and the corresponding strain rates were 10^−6^ s^−1^, 10^−5^ s^−1^, 10^−4^ s^−1^, 10^−3^ s^−1^, and 10^−2^ s^−1^, respectively, which were used to simulate quasi-static loading, vehicle load, and aircraft impact load (see Figure 2). A pre-load of 0.1 kN was applied to the specimen before loading, in order to obtain more stable experimental data. The tests were repeated three times at each rate, in order to ensure the accuracy of the test.

**Figure 3:**
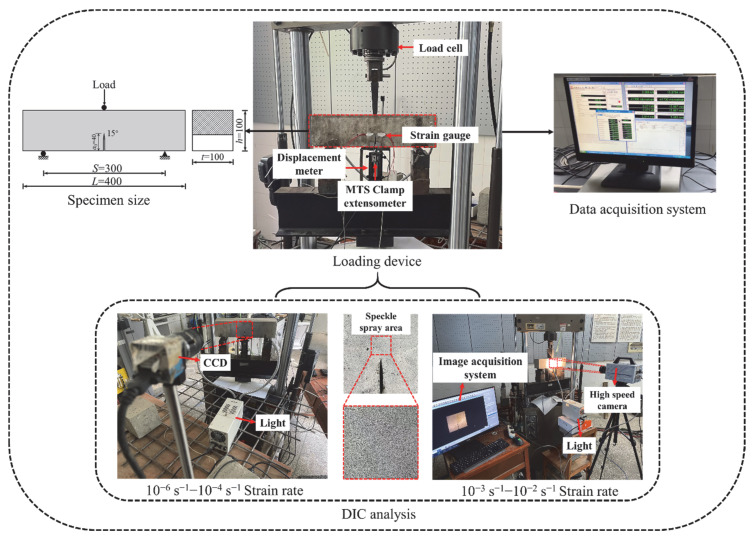
Diagram of the test device.

A pair of resistive strain gauges with a distance of 10 mm was arranged symmetrically at 10 mm from the crack tip of the specimen, in order to determine the crack initiation load *P*_ini_ during loading. The specimens were smoothed with a grinding machine before the test, ensuring that the strain gauge and the surface of the specimen fit flat. The crack mouth opening displacement (CMOD) of the specimen was measured using an MTS self-contained clip-on extensometer, with measuring range of 5 mm. The mid-span displacement was measured using a displacement sensor with a range of 10 mm. The above data were collected synchronously by a dynamic strain tester (DH 15202). DIC was used to monitor crack growth during loading. Before the test, a layer of white matte paint was sprayed on the crack propagation area of the specimen, following which a black speckle was sprayed. We adjusted the lens position, exposure, and focal length to obtain clear images. The image acquisition frequency was adjusted, according to the different loading rates. The NCORR image analysis software was used to calculate the displacement and strain of the specimen surface. The whole test device is shown in Figure 3.

## 3. Results and Discussions

### 3.1. Fracture Section

**Figure 4:**
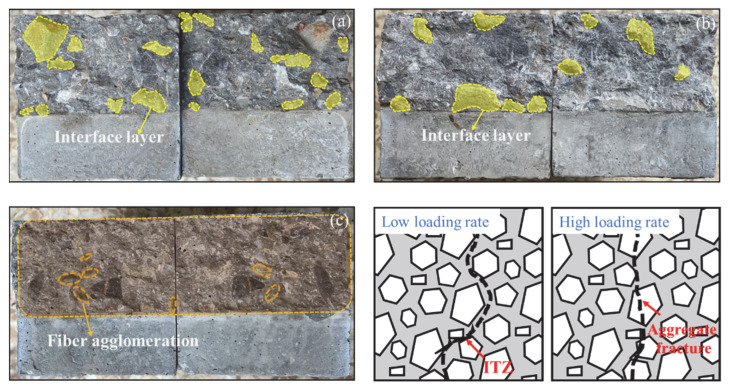
The failure sections of concrete at different loading rates: (**a**) 10^−6^ s^−1^; (**b**) 10^−4^ s^−1^; and (**c**) 10^−2^ s^−1^.

The fracture cross-sections of BFAPC at different strain rates are shown in Figure 4. At strain rates of 10^−6^ s^−1^ and 10^−4^ s^−1^, the crack propagation path of the specimen presented a certain deflection, and the fracture surface was rough with some partially intact aggregates. At strain rates of 10^−2^ s^−1^, the fracture surface of the specimen gradually became flat, and more and more coarse aggregates failed along the fracture surface. This is due to the existence of a large number of micro-cracks at the interface between the coarse aggregate and the mortar, which is the weakest area in the matrix [20]. At lower strain rates, the crack propagated from the interfacial transition zone (ITZ) of the aggregate–mortar. With an increase in the strain rate, the crack did not have enough time to pass through the ITZ. This caused it to pass directly through the coarse aggregate, making the fracture surface smoother. The same result was found in reference [20,29]. The strength of the aggregate is usually higher than the ITZ, which is one of the reasons for the increased fracture strength at high strain rates [23,30].

### 3.2. P-CMOD Curve

**Figure 5:**
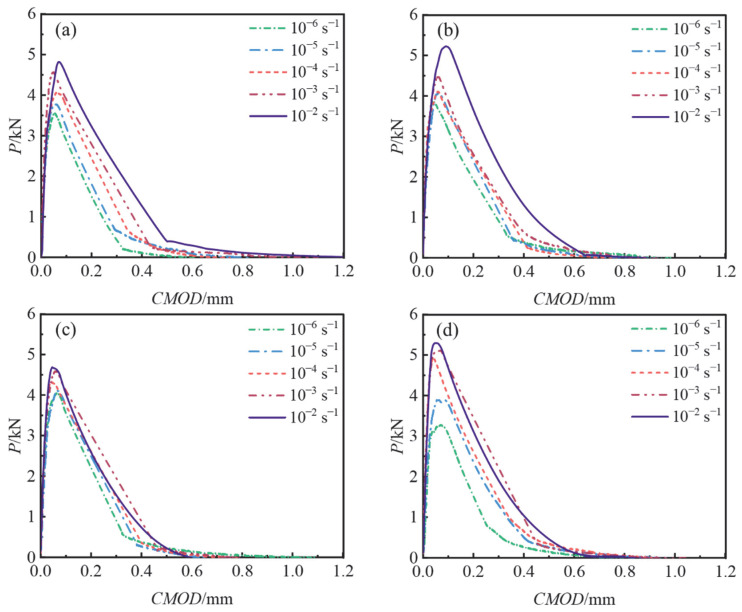
The *P–CMOD* curves under different loading rates: (**a**) OPC; (**b**) B-0.2; (**c**) B-0.4; and (**d**) B-0.6.

The changes in the *P–CMOD* curves of BFAPC at different strain rates are shown in Figure 5. According to the *P–CMOD* curve, the trends of the curves under different strain rates were basically the same. The fracture process of OPC presented an obvious brittle fracture effect at different strain rates, with the curve dropping rapidly after reaching the peak load. When the fiber content was 0.6%, the ductility performance of the specimens after reaching the instability extension stage was more obvious; this was due to the “bridging” effect of fibers in the matrix, which can inhibit the rapid propagation of cracks [11,31], thus improving the fracture toughness of the concrete. It was also found that the strain rate had a significant effect on the peak load of concrete with different fiber content. 

**Figure 6:**
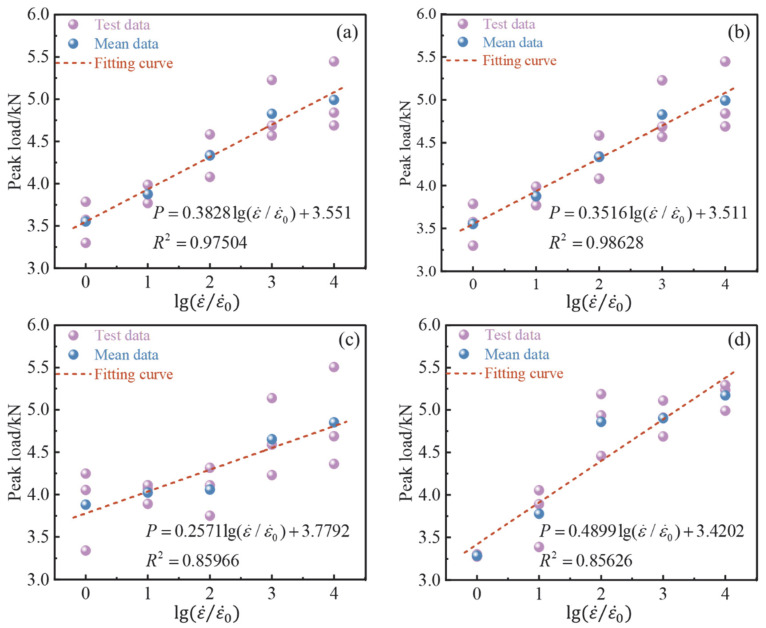
The relationship between the peak load and strain rate: (**a**) OPC; (**b**) B-0.2; (**c**) B-0.4; and (**d**) B-0.6.

Figure 6 shows the relationship between the strain rate and peak load. ${\dot{\varepsilon }}_{0}$ is the strain rate under quasi-static loading (here it is taken as 10^−6^ s^−1^); $\dot{\varepsilon }$ is the five strain rates selected in this paper (the same below). It is clear that the peak load was positively correlated with the logarithm of the relative strain rate; previous studies have also shown that the strain rate has a similar relationship with the peak load [32,33]. From the fitting curve, it can be seen that the peak load of concrete with different fiber content reached the maximum strain rate of 10^−2^ s^−1^, and BFAPC with 0.6% fiber content presented the highest peak load of 5.17 kN. In addition, the range of variation of peak load in each group was 40.5%, 38.8%, 25%, and 57.4%, respectively. In terms of the range of variation, the peak load of group B-0.6 had a higher strain rate sensitivity, while the strain rate sensitivity of group B-0.4 was relatively weak. Hillerborg [34] proposed the virtual crack theory, in which the peak fracture load of concrete is determined by its uniaxial tensile strength [19]. In addition, previous studies [30] have concluded that the uniaxial tensile strength of concrete increases with the increase of strain rate, namely strain rate effect. Therefore, due to the strain rate effect, the increase of the tensile strength of concrete leads to the increase of its peak fracture load. Section 3.1 explains the increase of fracture strength from a macroscopic fracture surface perspective.

**Figure 7:**
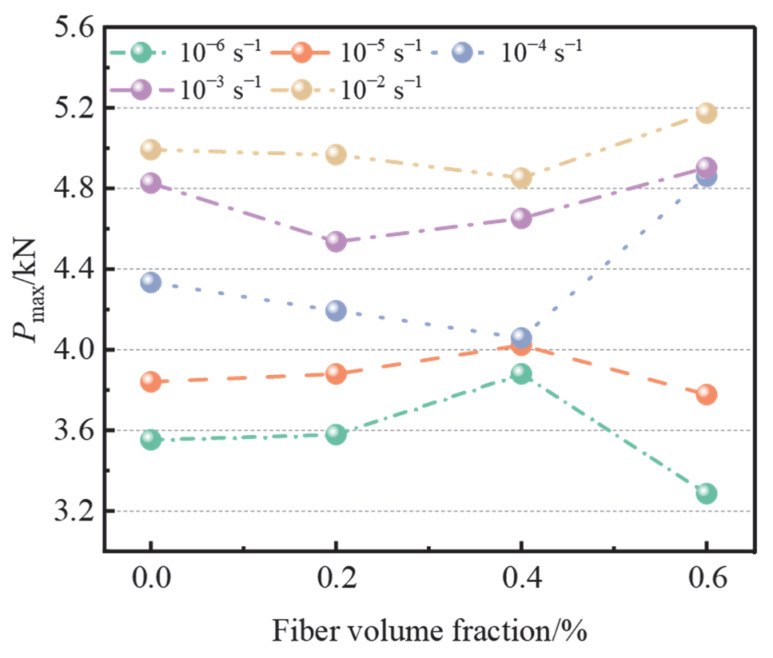
Peak loads under different fiber content.

The variation curves of peak load with different fiber content are shown in Figure 7. It can be seen that BF had a certain effect on the peak load. Compared with OPC, the peak load of BFAPC with 0.4% fiber content was the highest at strain rates of 10^−6^ s^−1^ and 10^−5^ s^−1^, which increased by 3.77% and 9.23%, respectively. However, the peak load of concrete at 0.6% fiber content decreased by 7.51% and 2.55%, respectively, indicating that this level of content has a negative effect at low strain rates. This may be due to the fiber more easily agglomerating at high dosages, which increases the weak interface within the matrix. In addition, the peak loads of BFAPC at strain rates of 10^−4^ s^−1^, 10^−3^ s^−1^, and 10^−2^ s^−1^ were reduced under 0.2% and 0.4% fiber content; however, the peak loads were increased by 12.14%, 3.72%, and 4.46% for 0.6% fiber content, respectively. It can be concluded that, under low strain rate, 0.4% fiber content can improve the fracture load of concrete, to a certain extent. Although the concrete with 0.6% fiber content had a negative effect on the peak fracture load at low strain rates, it presented a certain strengthening effect at high strain rates.

### 3.3. Initial Cracking Load

#### 3.3.1. Determination of *P*_ini_

For prefabricated cracked members, the determination of *P*_ini_ can be accomplished through the test curve method and strain gauge method [21,35,36]. The test curve method takes the turning point from linear to non-linear in the rising section of *P–CMOD* curve as $P_{\mathrm{ini}}^{c}$; however, the turning point is difficult to determine precisely. According to the literature [18,36], the load corresponding to a CMOD of 0.02 mm can be considered as the initial cracking load. In addition, the initial cracking load of BFAPC can also be determined by applying resistance strain gauges. The load value corresponding to the strain retraction point in the *P*-*ε* curve is chosen as $P_{\mathrm{ini}}^{s}$. The strain gauge method for determining the initial cracking load is sensitive to the strength of the material. It has good applicability for measuring the crack initiation load of high-strength concrete [37], making it a commonly used method for determining the initial cracking load. However, due to the differences in the adhesion between the strain gauges and the surface of the specimen, as well as the non-homogeneous material properties of concrete, the *P*_ini_ values measured in the same group may differ. The measured *P*_ini_ values are given in Table 3.

**Figure 8:**
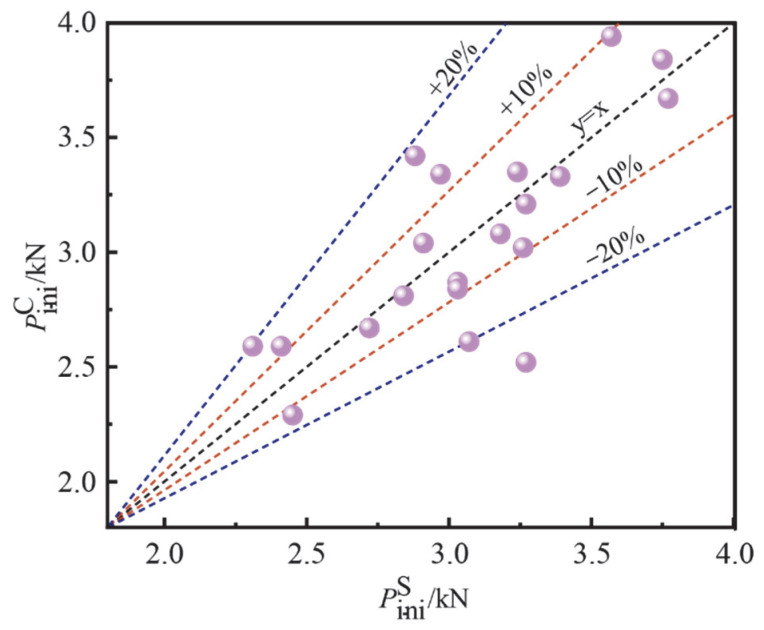
Comparison of the *P*_ini_ value obtained by the test curve method and the strain gauge method.

Figure 8 contrasts the *P*_ini_ measured by the strain gauge method and the test curve method, in order to verify the validity of the two methods. The black dashed line in the figure indicates y = x. The two red dashed lines indicate that the ratio of $P_{\mathrm{ini}}^{s}$ − $P_{\mathrm{ini}}^{c}$ to $P_{\mathrm{ini}}^{c}$ is ±10%. The two blue dashed lines indicate that the ratio of $P_{\mathrm{ini}}^{s}-P_{\mathrm{ini}}^{c}$ to $P_{\mathrm{ini}}^{c}$ is ±20%. It can be seen from the figure that 70% of the initial cracking load measured by both methods fell within the ±10% range, and 95% of the data points fell within the ±20% range. Overall, the results indicate that the difference between the cracking load measured by the strain gauge method and the test curve method was not significant. Therefore, it is appropriate to adopt the load corresponding to CMOD = 0.02 mm as the initial cracking load. By using the two methods for determining the initial cracking load, the test results can be cross-validated.

#### 3.3.2. Effect of Loading Rate and Fiber Content on Initial Cracking Load

**Figure 9:**
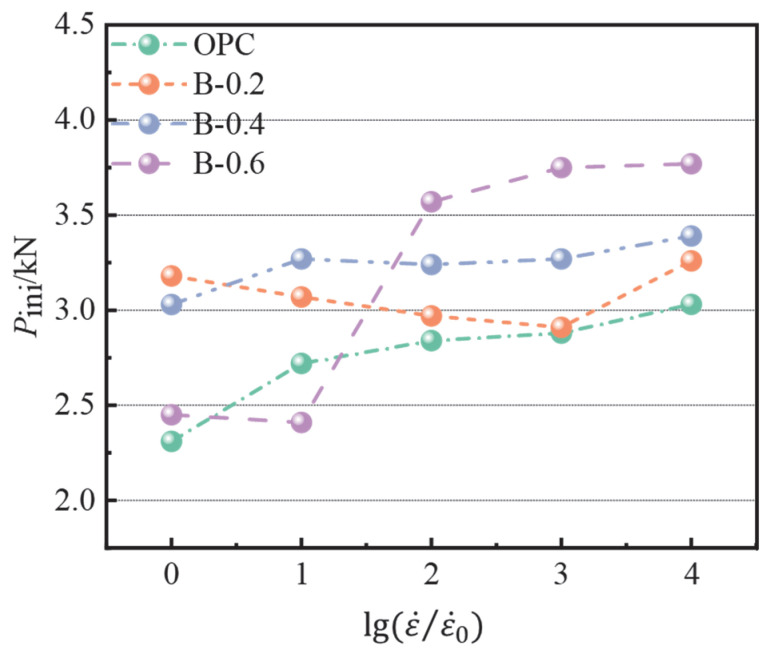
Relationship between the initial cracking load and strain rate.

Figure 9 shows the relationship between the strain rate and initial cracking load. From the figure, it can be seen that there was a certain strain rate effect of the initial cracking load, but there was no obvious pattern between it and the strain rate. Compared with the initial cracking load when the strain rate was 10^−6^ s^−1^, the initial cracking load of each group at 10^−2^ s^−1^ was increased (by 31.2%, 2.5%, 11.9%, and 53.9%, respectively). It can be seen that the strain rate had the most significant effect on the initial cracking load of the OPC and B-0.6 groups. It is worth noting that, at 0.6% fiber content, the initial cracking load of concrete changed abruptly during the transition from 10^−5^ s^−1^ to 10^−4^ s^−1^ strain rate (increased by 48.13%), demonstrating that this content had the highest initial cracking load at a higher strain rate, while it did not have an effective role at low strain rate. The sensitivity of the initial cracking load to the loading rate was higher for this content.

### 3.4. Double K Fracture Toughness

Xu and Reinhardt [38,39] originally proposed the double-K fracture model. Hu et al. [40] have found that different strain rates have a certain effect on the double-K fracture toughness, and the double-K fracture model is also applicable at high strain rates, such as 10^−3^ s^−1^ and 10^−2^ s^−1^. To ensure the accuracy of the calculation of the fracture characteristic parameters of the three-point bending beam, the influence of the self-weight factor is considered in the calculation [41]. The crack initiation toughness $K_{\mathrm{Ic}}^{\mathrm{ini}}$, instability toughness $K_{\mathrm{Ic}}^{\mathrm{un}}$, and critical effective crack length $a_{c}$ are calculated as follows:

(1) $$K_{\mathrm{Ic}}^{\mathrm{ini}}=\frac{1.5(P_{\mathrm{ini}}+mg/2\times 10^{-2})\times 10^{-3}\times S\times \sqrt{a_{0}}}{th^{2}}f\left(\alpha_{0}\right),$$

(2) $$f\left(\alpha_{0}\right)=\frac{1.99-\alpha_{0}\left(1-\alpha_{0}\right)\left(2.15-3.93\alpha_{0}+2.7{\alpha_{0}}^{2}\right)}{\left(1+2\alpha_{0}\right){\left(1-\alpha_{0}\right)}^{\frac{3}{2}}},\;\alpha_{0}=\frac{a_{0}}{h},$$

(3) $$K_{\mathrm{Ic}}^{\mathrm{un}}=\frac{1.5(P_{\max}+mg/2\times 10^{-2})\times 10^{-3}\times S\times \sqrt{a_{c}}}{th^{2}}f\left(\alpha_{c}\right),$$

(4) $$f\left(\alpha_{c}\right)=\frac{1.99-\alpha_{c}\left(1-\alpha_{c}\right)\left(2.15-3.93\alpha_{c}+2.7{\alpha_{c}}^{2}\right)}{\left(1+2\alpha_{c}\right){\left(1-\alpha_{c}\right)}^{\frac{3}{2}}},\;\alpha_{c}=\frac{a_{c}}{h},$$

where $P_{\mathrm{ini}}$ is the test measured cracking load (kN); $P_{\max}$ and ${\mathrm{CMOD}}_{c}$ are the maximum measured instability load and the corresponding crack mouth opening displacement (μm), respectively; m is the span mass of the specimen (kg); the acceleration due to gravity *g* is taken as 9.81 m/s^2^; S is the span (m); *t* is the specimen width (m); *h* is the specimen height (m); and *a*_0_ is the prefabricated crack height (m).

(5) $$a_{c}=\frac{2}{\pi }\left(h+h_{0}\right)\arctan\sqrt{\frac{{tE\times CMOD}_{c}+\Delta CMOD}{32.6\left(P_{\max}+\Delta P\right)}-0.1135}-h_{0},$$

where $h_{0}$ is the thickness of the fixed clip-type extensometer blade (m; as it is fixed on the side, here it is taken as 0); E is the initial modulus of elasticity; and Δ*P* and Δ*CMOD* are the self-weight correction formulae for *P* and *CMOD*, respectively.

(6) $$E=\frac{1}{tc_{i}}[3.70+32.60\;\tan^{2}(\frac{\pi }{2}\frac{a_{0}+h_{0}}{h+h_{0}})],\;c_{i}=\frac{CMOD_{i}}{P_{i}},$$

(7) $$\Delta P=\frac{mg}{2}\times 10^{-2},$$

(8) $$\Delta CMOD=\frac{\Delta P}{tE}[3.70+32.60\;\tan^{2}(\frac{\pi }{2}\frac{a_{0}+h_{0}}{h+h_{0}})].$$

For the calculation of the initial modulus of elasticity *E*, *c*_i_ is the initial flexibility (μm/kN), which is generally taken as the ratio of *CMOD*_ini_ to *P*_ini_. The ratio is brought into the formula to calculate the cutline modulus of elasticity *E*_1_ at the point, while the initial segment of the actually measured *P*-*CMOD* curve is not a relatively stable linear segment. Another way to calculate the tangent elastic modulus *E*_2_ is to perform a linear fit to the initial segment of the curve, using its slope *k* instead of 1/*c*_i_. It has been found that there is little difference in the double K fracture toughness measured using *E*_1_ and *E*_2_ [41]. The double-K fracture toughness calculated using *E*_1_ for this test is shown in Table 4.

**Figure 10:**
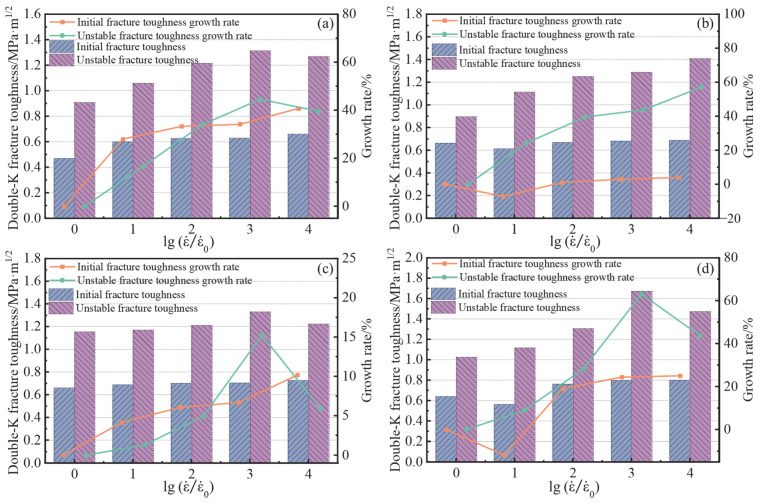
The relationship between the double K fracture toughness and strain rate: (**a**) OPC; (**b**) B-0.2; (**c**) B-0.4; and (**d**) B-0.6.

The effect law of different strain rates on the double K fracture toughness of concrete is shown in Figure 10. It can be clearly seen that the strain rate has some effect on the double K fracture toughness. In the strain rate range of this study, according to the growth rate of initial fracture toughness, the strain rate effect in the OPC group was the most obvious, which was increased by 40.72%, and the maximum increase in the B-0.6 group was 25%, while the strain rate effect of initial fracture toughness was not obvious at other fiber contents. For the unstable fracture toughness $K_{\mathrm{Ic}}^{\mathrm{un}}$, the maximum increase of each group was 44.56%, 57.25%, 15.25%, and 63.12%. Except for the B-0.2 group, where the instability toughness increased with increasing strain rate, the other groups showed a weak downward trend at a strain rate of 10^−2^ s^−1^. In the studies of Chen [19] and Hu [40], it was found that, when the strain rate is in the range 10^−6^ s^−1^–10^−3^ s^−1^, there exists a positive correlation between the unstable fracture toughness and strain rate. However, the unstable fracture toughness no longer increased at a strain rate of 10^−2^ s^−1^. According to Equation (3), the magnitude of unstable fracture toughness is related to *P*_max_ and *a*_c_. As *P*_max_ was the highest at a strain rate of 10^−2^ s^−1^, the weakening of the critical fracture length *a*_c_ at the strain rate of 10^−2^ s^−1^ appears to affect the increase in instability toughness.

The initial fracture toughness $K_{\mathrm{Ic}}^{\mathrm{ini}}$ expresses the ability of the member to resist crack initiation, while unstable fracture toughness $K_{\mathrm{Ic}}^{\mathrm{un}}$ expresses the ability of the member to resist crack expansion under maximum load. By comparing the calculated results in Table 4, it was found that the maximum initial fracture toughness of B-0.2 and B-0.4 groups was 0.662 and 0.687 MPa·m^1/2^ at strain rates of 10^−6^ s^−1^ and 10^−5^ s^−1^, respectively, while the maximum initial fracture toughness of group B-0.6 was 0.726, 0.796, and 0.8 MPa·m^1/2^ at strain rates of 10^−4^ s^−1^, 10^−3^ s^−1^, and 10^−2^ s^−1^, respectively. This is consistent with the relevant rules of *P*_ini_ in Section 3.3.2, as the initial fracture toughness is mainly determined by *P*_ini_, and the difference in specimen quality only changes the cracking toughness by a small amount between different doping levels. As the initial fracture toughness is influenced by the ITZ strength [42], the bridging effect of BF increases the strength of ITZ during the crack initiation stage, which inhibits the rapid development of initiating cracks, to some extent. In addition, the maximum unstable fracture toughness of B-0.4 and B-0.6 groups was 1.169 and 1.672 MPa·m^1/2^ at strain rates of 10^−5^ s^−1^ and 10^−3^ s^−1^, respectively. With 0.4% fiber content, BF can effectively improve the ability of concrete to resist instability fractures at low strain rates. Meanwhile, the 0.6% fiber content can improve the load-bearing capacity of concrete at higher strain rates. The observed increase in fracture toughness is not only related to the amount of BF content, but also to the distribution of fibers in the matrix and the bond degree between fiber and concrete matrix [43]. Therefore, more research is necessary to determine why adding BF can enhance concrete’s resistance to crack instability expansion at different strain rates.

### 3.5. Fracture Energy and Ductility Index

The fracture energy *G*_F_ is the total energy consumed by the material to form a fracture zone per unit area [44]. It reflects the ability of a material to resist crack growth and is yet another crucial factor to consider while evaluating the fracture performance of BFAPC. It can be determined in terms of the area under the load–deflection curve (*P-δ*) during the three-point bending fracture of the material [31]. The influence of specimen weight is considered in the calculation, expressed as follows:

(9) $$G_{F}=\frac{W_{0}+mg\delta_{c}}{A_{lig}},$$

where *W*_0_ is the total work performed by the external force (N·m); *m* is the mass of the beam (kg); *g* is equal to 9.8 m/s^2^; *δ*_c_ is the final mid-span deformation (mm); and $A_{lig}$ is the area of the fracture section (m^2^).

Ductility index is a parameter, proposed by Chiaia [45], used to measure the cracking deformation of concrete. It reflects the brittleness of the material: the smaller the index, the more brittle the material. The formula for calculating the ductility index is:

(10) $$D_{u}=G_{F}/P_{\max},$$

where *P*_max_ is the peak load (kN).

**Figure 11:**
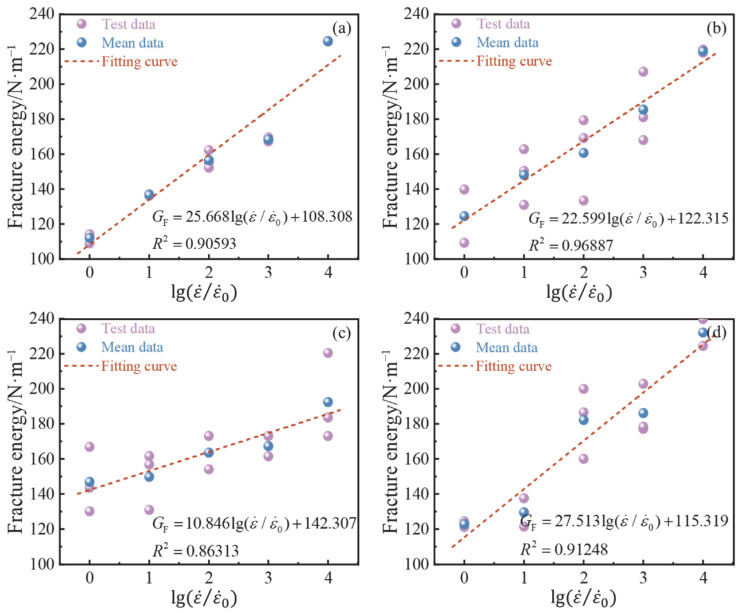
The relationship between the fracture energy and strain rate: (**a**) OPC; (**b**) B-0.2; (**c**) B-0.4; and (**d**) B-0.6.

Table 4 displays the fracture energy determined for each group. Figure 11 shows the change trend of fracture energy of BFAPC at different strain rates. It is obvious that the fracture energy also presented a certain strain rate effect. There was a positive correlation between the fracture energy and the relative strain rate’s logarithm. The highest fracture energy of each group was 224.53, 218.87, 192.40, and 232.14 N·m^−1^ when the strain rate reached 10^−2^ s^−1^. This was due to the fact that, under a high strain rate, the concrete directly passes through the coarse aggregate when it is destroyed, which increases the fracture strength and ultimately requires more energy to cause concrete damage. The fracture section in Section 3.1 also demonstrates this fact. In addition, as the rate rises, the formation of microcracks is also another important factor affecting the strain rate effect of fracture energy [46]. Relative to the high loading rate of 10^2^–10^3^ mm/s, Chen [20] and Zhang [47] have suggested that this rate effect is less sensitive at low loading rates, such as 10^−1^ mm/s.

**Figure 12:**
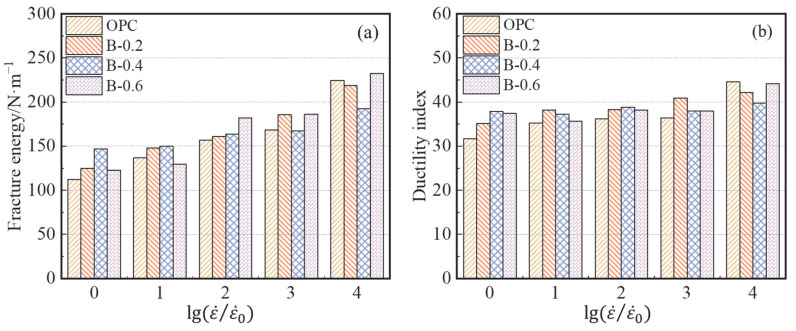
(**a**) The fracture energy and (**b**) ductility index of BFAPC with different fiber contents.

As shown in Figure 12a, the addition of BF has a significant effect on the fracture energy of concrete. Compared with the fracture energy of OPC, the failure of BFAPC requires more energy. At strain rates of 10^−6^ s^−1^ and 10^−5^ s^−1^, the highest fracture energy of B-0.4 group was 146.90 and 149.82 N·m^−1^, respectively, whereas the highest fracture energy of B-0.6 group was 182.17, 186.12, and 232.14 N·m^−1^ at strain rates of 10^–4^ s^−1^, 10^–3^ s^−1^, and 10^−2^ s^−1^, respectively. The reason for this is that the fracture damage of concrete requires additional energy to overcome the adhesive force between the fiber and matrix, as well as the energy consumed when the BF is pulled out. Thus, the toughness of BFAPC is improved; this higher toughness indicates that the specimen has a higher resistance to failure, which is also reflected in the relatively flat curve of the tail section of the *P–CMOD* curve shown in Section 3.2. Li et al. [10] found that a higher fiber volume content led to a greater number of fibers involved in crack resistance in the fracture process zone (FPZ) and, consequently, a greater amount of energy required to damage the concrete. From the above conclusions, the energy required for the fracture of fiber-reinforced concrete is also related to the strain rate the concrete is subjected to. As shown in Figure 12b, the ductility of concrete was only slightly improved or even decreased with the addition of BF. This phenomenon was also observed during the experiment. It can be concluded that, although the toughness of concrete will be improved by adding BF, the ductility may be weakened at high strain rates.

**Figure 13:**
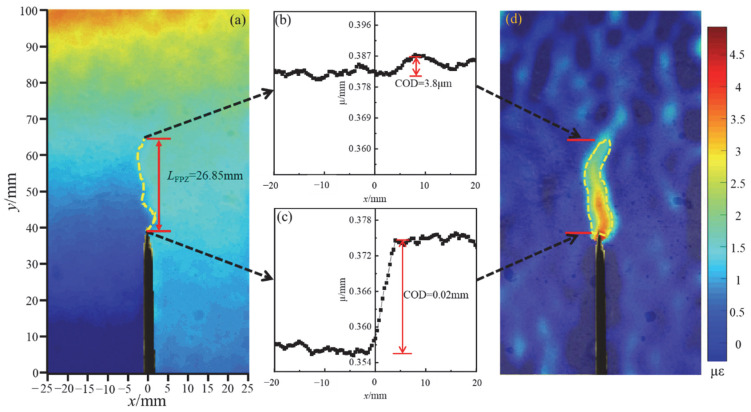
The BFAPC with 0.6% fiber content at strain rate of 10^−6^ s^−1^ at peak load: (**a**) COD contour; (**b**) COD pattern at y = 40 mm; (**c**) COD pattern at y = 66.85 mm; and (**d**) strain contour.

**Figure 14:**
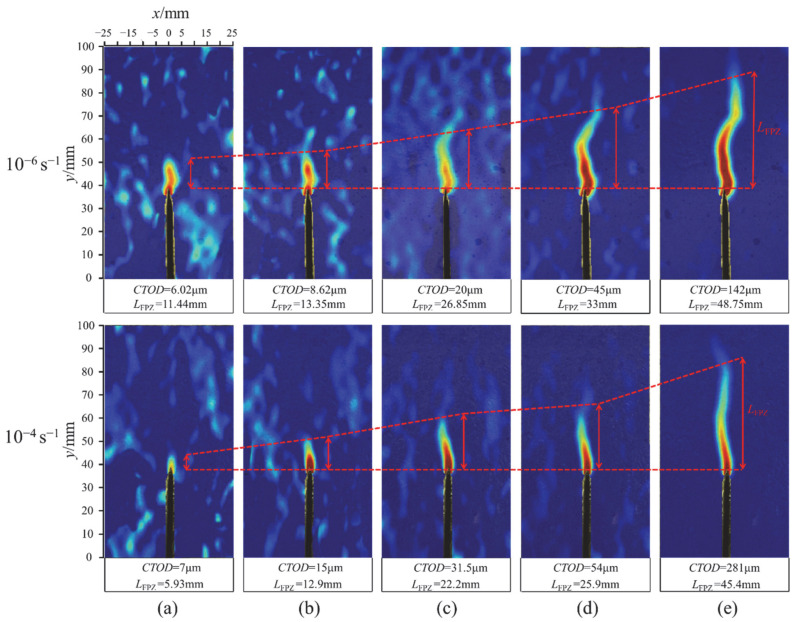
The strain contours of 0.6% BFAPC at strain rate of 10^−6^ s^−1^ and 10^−4^ s^−1^: (**a**) Pre-60%*P*_max_; (**b**) pre-80%*P*_max_; (**c**) *P*_max_; (**d**) post-80%*P*_max_; and (**e**) post-20%*P*_max_.

Compared with OPC, it can be seen that the fracture energy of 0.6% BFAPC had little improvement or was even decreased at a strain rate of 10^−6^ s^−1^ or 10^−5^ s^−1^, while its fracture energy at a strain rate of 10^−4^ s^−1^ or higher strain rate was the highest. Therefore, in order to obtain a more intuitive difference of strain rate effect in group B-0.6, the change rule of crack length and crack tip opening displacement (CTOD) throughout the BFAPC fracture process was examined by DIC. The displacement and strain contour diagrams at the peak load at 10^−6^ s^−1^ strain rate are shown in Figure 13a,d, respectively, while the lateral displacement changes at y = 40 mm and y = 66.85 mm are shown in Figure 13b,c, respectively. The crack opening displacement (COD) value is equal to the difference in displacement between the start and end points of the jump, and the generation of microcracks leads to the fluctuation of lateral displacement. Referring to the relevant literature [31,48], the location where the COD is about 5 μm is defined as the crack tip. Finally, the CTOD and crack length can be determined. Based on comprehensive analysis of strain contours at 10^−6^ s^−1^ and 10^−4^ s^−1^ strain rate, Figure 14a–e show the strain contour maps at pre-60% *P*_max_, pre-80% *P*_max_, *P*_max_, post-80% *P*_max_, and post-20% *P*_max_ stages at 10^−6^ s^−1^ and 10^−4^ s^−1^ strain rate, respectively. The larger the tension displacement of COD, the higher the consumed energy and peak load. Additionally, the results of pertinent research by Ma [21] and Zhu [23] have indicated that the fracture length and opening displacement increase with loading rate. BFAPC with 0.6% content had a smaller CTOD value but a longer crack propagation length at a strain rate of 10^−6^ s^−1^. This means that the fracture energy at this strain rate is low and the specimen is susceptible to failure, as the spreading of cracks could not be effectively contained by the fiber. However, the CTOD is greater and necessitates more energy consumption at a strain rate of 10^−4^ s^−1^. During the gradual upward expansion of the crack, the incorporated fibers inhibit crack extension. This explains the result that 0.6% BF content was more suitable at higher strain rates from another perspective.

### 3.6. Effects of Loading Rates on Crack Propagation

**Figure 15:**
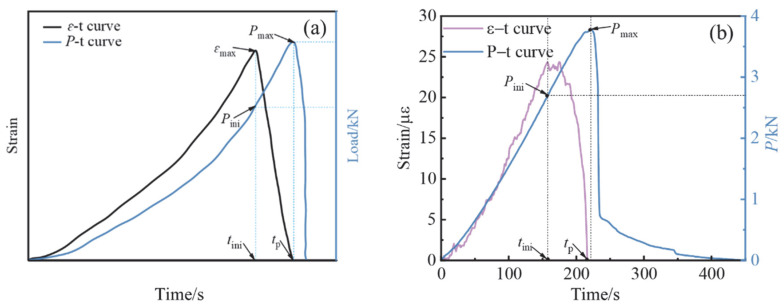
Variation diagram of strain and load with time: (**a**) schematic; and (**b**) experimental result.

The critical effective crack length *a*_c_ is an important parameter reflecting the amount of crack expansion at the critical instability condition. The performance of the crack propagation in FPZ can be quantitatively described by analyzing the critical crack growth rate. The literature [40] provides the formula for the critical crack expansion rate as:

(11) $$v_{c}=\frac{a_{c}-a_{0}}{t_{p}-t_{\mathrm{ini}}},$$

where *t_i_*_ni_ is the time corresponding to the maximum strain measured by the strain gauge at the crack tip and *t*_p_ is the time corresponding to the time when the specimen reaches the maximum load *P*_max_. The crack initiation time *t*_ini_ and the time to peak load *t*_p_ can be determined from the strain and load time variation curves. The schematic is shown in Figure 15a. Figure 15b depicts the relevant experimental results obtained in this paper. The figure makes it simple to determine *t*_ini_ and *t*_p_.

**Figure 16:**
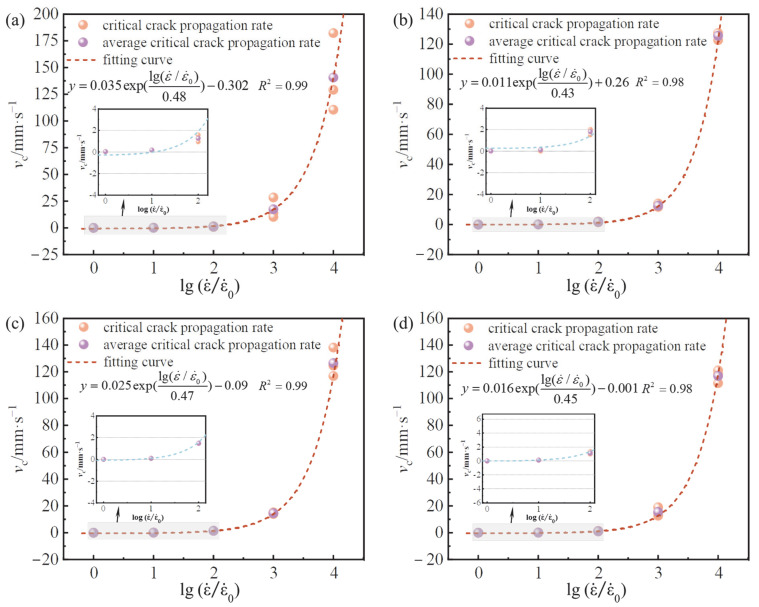
Critical crack propagation velocities at various loading rates: (**a**) OPC; (**b**) B-0.2; (**c**) B-0.4; and (**d**) B-0.6.

Figure 16 shows the relationship between the critical crack growth rate and strain rate under different BF contents. The crack extension rate grew slowly for strain rates of 10^−6^ s^−1^–10^−4^ s^−1^, while the extension rate increased significantly after 10^−3^ s^−1^. Similar results were achieved by Pyo [49] and Ngo [50]. Chen et al. [20] studied the crack growth rate of large-size OPC specimens under the loading rate of 0.0005–0.5 mm /s. It was found that his research results are basically within the range of the research results in this paper, which further confirms that the research results in this paper are valid. However, the difference between the specimen size and that of this paper may have a certain impact on the critical crack growth rate. The critical crack growth rate was no more than 1.81 mm/s when the strain rate was between 10^−6^ s^−1^ and 10^−4^ s^−1^. John [51] pointed out that, when the crack growth rate was on the order of 1 mm/s, the crack would expand under static conditions. If the crack expansion rate increases to a higher order of magnitude, the crack will expand at an approximate dynamic rate. Therefore, the crack extension rate in this test at 10^−4^ s^−1^ was on the same order of magnitude as 1 mm/s. It can be approximated that, when the strain rate is no higher than 10^−4^ s^−1^, the critical crack expands in the static state. At a strain rate not exceeding 10^−4^ s^−1^, it is anticipated that the fracture growth rate of BFAPC will be no greater than 2 mm/s. The maximum crack growth rate can reach 17.5 mm/s and 140 mm/s when the strain rate is 10^−3^ s^−1^ and 10^−2^ s^−1^, respectively. In this case, it can be determined that the crack is expanding at a dynamic rate. According to the fitted curve, the fracture growth rate increases exponentially as the strain rate rises, supporting the research findings of Hu [40]. This is useful for forecasting the BFAPC crack growth rate under various strain rates.

**Figure 17:**
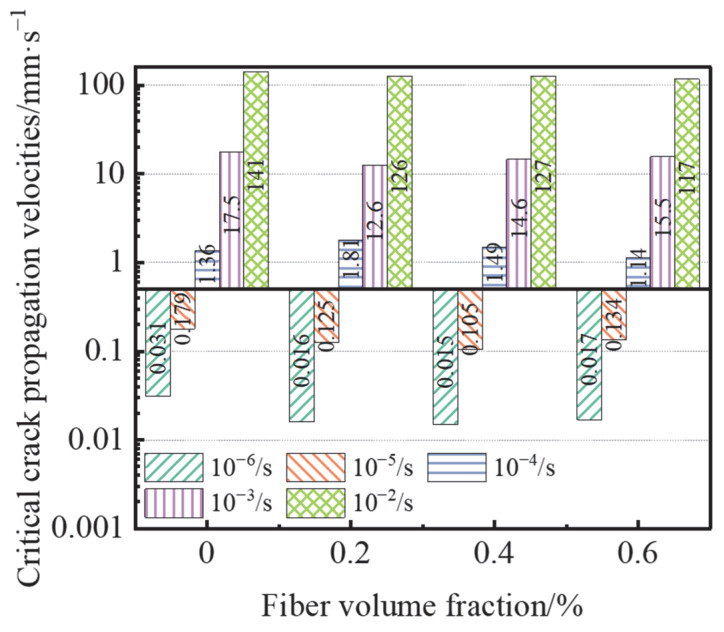
Effect of fiber content on critical crack propagation velocities.

Figure 17 depicts the impact of various BF addition on the critical fracture growth rate of concrete. Ngo [50] and Li [52] demonstrated that reinforcements significantly affected on the propagation of crack in ultra-high-performance fiber-reinforced concrete (UHPFRC) based on the crack growth rate. Compared with OPC, the crack extension rate was reduced by 51.6% and 41.3% for the B-0.4 group at strain rates of 10^−6^ s^−1^ and 10^−5^ s^−1^, respectively, this indicates that this dose had the best effect, in terms of suppressing cracks in static expansion; the crack extension rate was reduced by 28% for the B-0.2 group at strain rates of 10^−3^ s^−1^; meanwhile, the fiber content of 0.6% played a better role in suppressing crack expansion of concrete (reduced by 16.18% and 17.02%, respectively) at a strain rate of 10^−4^ s^−1^ and 10^−2^ s^−1^. No obvious pattern was determined between the fiber admixture and the critical crack expansion rate at the same strain rate. In conclusion, the incorporation of BF inhibited the crack expansion of concrete and reduced the critical crack expansion rate.

## 4. Conclusions

In this study, we considered a range of strain rates under different working conditions and conducted a fracture test of airport pavement concrete with different BF contents. The peak load, initial cracking load, double-K fracture toughness, fracture energy, critical crack growth rate, and other metrics were used to systematically assess how the strain rate and fiber content affect the concrete’s fracture performance. Our main conclusions are summarized as follows:

1. The fracture cross-section of the BFAPC exhibited distinct fracture characteristics under different strain rates. At a lower strain rate, the cracks began to expand along the ITZ and gradually extended upward, with some aggregates exposed on the surface. At high strain rate, the crack directly passed through the coarse aggregate, and the surface was relatively flat.
2. The peak load of OPC and BFAPC increased with an increase in strain rate, showing an obvious strain rate effect. When the strain rate increases from 10^−6^ s^−1^ to 10^−2^ s^−1^, the range of increment of peak load in each group was 40.5%, 38.8%, 25%, and 57.4%, respectively. Although the strain rate sensitivity of peak load in group B-0.6 was the highest, there was no clear relationship between fiber content and the strain rate sensitivity.
3. The double K fracture toughness and fracture energy both exhibited strain rate effects. In the selected range of strain rate, the initial fracture toughness showed the largest increase in the OPC group (40.72%); the unstable fracture toughness showed the largest increase in the B-0.6 group (63.12%). With an increase in strain rate, the fracture energy of OPC and BFAPC increased. Compared with OPC, the addition of BF improved the fracture toughness of airport pavement concrete at different strain rates, to a certain extent.
4. BF can bridge BFPAC more effectively at higher strain rate to prevent its crack propagation based on the variation law of crack propagation length and CTOD of group B-0.6 at different strain rates.
5. The critical crack expansion rate *v*_c_ of both OPC and BFAPC increased slowly with an increase in strain rate between 10^−6^ s^−1^ and 10^−4^ s^−1^, with none of them exceeding 1.81 mm/s. The expansion rate increased significantly after 10^−3^ s^−1^, presenting an exponential increase. The addition of BF reduced the crack growth rate of concrete, to some extent.
6. The initial cracking load measured by test curve method (*CMOD*_ini_ = 0.02 mm) was in good agreement with that obtained with the Strain gauge method. Therefore, the test curve method can be used as a convenient and inexpensive method to obtain the crack initiation load; however, its accuracy in other applications requires further investigation.

## Figures and Tables

**Table 1:** Mixing ratio of airport pavement concrete.

No.	Fiber Length/mm	Volume Fraction/%	Mixing Proportion/kg·m^−3^							
			Cement	Fly Ash	Slag	Water	Sand	Coarse Aggregate	Fine Aggregate	Water Reducing Agent
OPC	-	-	307.74	67.44	46.38	155.97	565.87	886.53	433.84	2.108
B-0.2	12	0.2	307.74	67.44	46.38	155.97	565.87	886.53	433.84	2.250
B-0.4	12	0.4	307.74	67.44	46.38	155.97	565.87	886.53	433.84	2.500
B-0.6	12	0.6	307.74	67.44	46.38	155.97	565.87	886.53	433.84	2.633

**Table 2:** Physical and mechanical indices of basalt fiber.

Length/mm	Diameter/μm	Density/ (g∙cm^−3^)	Elasticity Modulus /GPa	Tensile Strength /MPa	Fracture Ductility Rate/%
12	13.8	2.699	95–110	3500–4500	3.1

**Table 3:** Statistical table of fracture loads.

No.	Strain Rate/(s^−1^)	Strain Gauge Method		Test Curve Method		*P*_max_/kN	*CMOD*_c_/μm
		$P_{\mathrm{ini}}^{s}/\mathrm{kN}$	*CMOD*_ini_/μm	$P_{\mathrm{ini}}^{c}/\mathrm{kN}$	*CMOD*_ini_/μm		
OPC	10^−6^	2.31	18.85	2.59	19.98	3.553	50.89
	10^−5^	2.72	21.04	2.67	20.04	3.877	53.44
	10^−4^	2.84	20.57	2.81	20.02	4.334	61.80
	10^−3^	2.88	14.98	3.42	20.14	4.827	57.47
	10^−2^	3.03	21.70	2.87	20.17	4.992	59.60
B-0.2	10^−6^	3.18	21.12	3.08	19.90	3.579	36.50
	10^−5^	3.07	25.52	2.61	20.29	3.88	59.69
	10^−4^	2.97	15.49	3.34	20.44	4.193	60.83
	10^−3^	2.91	18.58	3.04	20.16	4.536	59.14
	10^−2^	3.26	24.15	3.02	20.35	4.967	79.58
B-0.4	10^−6^	3.03	22.88	2.84	19.94	3.881	59.55
	10^−5^	3.27	28.92	2.52	20.01	4.023	66.78
	10^−4^	3.24	18.83	3.35	19.95	4.059	44.58
	10^−3^	3.27	20.56	3.21	19.90	4.652	58.28
	10^−2^	3.39	20.36	3.33	19.87	4.852	47.11
B-0.6	10^−6^	2.45	24.63	2.29	20.15	3.286	68.68
	10^−5^	2.41	19.47	2.59	20.15	3.778	58.48
	10^−4^	3.57	17.24	3.94	20.08	4.86	41.24
	10^−3^	3.75	19.32	3.84	20.06	4.903	66.42
	10^−2^	3.77	20.81	3.67	20.13	5.173	55.51

**Table 4:** Related fracture parameters of concrete under different loading rates.

No.	Strain Rate/(s^−1^)	$K_{\mathrm{Ic}}^{\mathrm{ini}}$ /(MPa·m^1/2^)	$\mathrm{Gain}\;\mathrm{Ratio}\;\mathrm{of}\;K_{\mathrm{Ic}}^{\mathrm{ini}}$	$K_{\mathrm{Ic}}^{\mathrm{un}}$ /(MPa·m^1/2^)	$\mathrm{Gain}\;\mathrm{Ratio}\;\mathrm{of}\;K_{\mathrm{Ic}}^{\mathrm{un}}$	G_F_/(N·m^−1^)	D_u_
OPC	10^−6^	0.469	0	0.908	0	112.06	31.61
	10^−5^	0.600	27.93	1.059	16.61	136.67	35.24
	10^−4^	0.625	33.26	1.215	33.86	156.56	36.23
	10^−3^	0.629	34.12	1.313	44.56	168.41	36.39
	10^−2^	0.660	40.72	1.267	39.54	224.53	44.55
B-0.2	10^−6^	0.662	0	0.896	0	124.54	35.17
	10^−5^	0.614	−7.26	1.113	24.24	148.09	38.15
	10^−4^	0.669	1.05	1.250	39.51	160.65	38.30
	10^−3^	0.681	2.95	1.287	43.71	185.42	40.93
	10^−2^	0.688	3.91	1.409	57.25	218.87	42.16
B-0.4	10^−6^	0.660	0	1.154	0	146.90	37.88
	10^−5^	0.687	4.14	1.169	1.3	149.82	37.18
	10^−4^	0.700	6.06	1.211	4.94	163.59	38.80
	10^−3^	0.704	6.67	1.330	15.25	167.29	37.94
	10^−2^	0.727	10.15	1.222	5.89	192.40	39.71
B-0.6	10^−6^	0.640	0	1.025	0	122.87	37.40
	10^−5^	0.564	−11.88	1.117	8.98	129.54	35.61
	10^−4^	0.762	19.06	1.306	28.1	182.17	38.19
	10^−3^	0.796	24.38	1.672	63.12	186.12	37.94
	10^−2^	0.800	25	1.472	43.61	232.14	44.13

## Data Availability

Not applicable.
